# Occurrence and distribution of meso- and macroplastics in the water, sediment, and fauna of the Nile River, Egypt

**DOI:** 10.1007/s10661-023-11696-7

**Published:** 2023-08-31

**Authors:** Yasmine A. M. Hassan, Ahmed E. A. Badrey, Alaa G. M. Osman, Aldoushy Mahdy

**Affiliations:** Department of Zoology, Faculty of Science, Al-Azhar University (Assiut Branch), Assiut, 71524 Egypt

**Keywords:** Macroplastic, *Oreochromis*, *Clarias*, *Procamparus*, Nile River, Upper Egypt

## Abstract

The present study described the most recent findings concerning the abundance and distribution of plastic in water, sediment, and fauna in the Nile River of Upper Egypt as an interesting research point. The findings revealed that plastics were abundant in the water, sediments, fish, and crayfish throughout the sites. The Nagaa Hammadi site has the highest abundance of meso- and macroplastics in its water and sediment. African catfish had the highest abundance of meso- and macroplastics compared to the other species, while Nile tilapia had no meso- or macroplastics in its alimentary canal or gills in all sites. The Edfu site has the highest abundance of mesoplastics in the alimentary canals of African catfish, while the Nagaa Hammadi site has the highest abundance of mesoplastics in the gills, and macroplastics appeared only in the alimentary canal of African catfish from the El-wasta site. Only mesoplastics were found in the crayfish’s alimentary canal, with the Nagaa Hammadi site having the highest abundance. No macroplastics were detected in the crayfish’s gills or alimentary canal. Additionally, this work lets us understand how plastics behave in freshwater environments, and it is a step toward decision-makers taking appropriate measures to reduce their risk.

## Introduction

One of the main contributors to the so-called novel entities, which are being referred to as a “chemical intensification” as a result of the increasing global chemical production and the expanding global distribution of chemical products or consumer goods, are plastic polymers that degrade to microplastics (Martínez et al., 2021). Plastic, a lightweight and long-lived material, has significantly increased the environmental risk (Thompson et al., 2009). Since the 1950s, synthetic polymers have been produced and used effectively (Geyer et al., 2017). Currently, the global production of plastic is estimated to be ∼367 million metric tonnes in 2020 (Tiseo, 2022). Only 9% and 12% of plastic waste have been recycled and incinerated, while the remaining 79% of plastic waste is in landfills (UN Environment, 2018). The interest in studying plastic pollution in aquatic ecosystems began in recent years and has continued to grow until now. In a study carried out by Kasavan et al. (2021) to explore research trends regarding plastic pollution in water ecosystems between 2000 and 2020, a total of 2182 papers on plastic pollution in water ecosystems were identified. This found that, as opposed to freshwater ecosystem–focused research, most earlier studies in phase I (2000–2006) and phase II (2007–2013) concentrated on plastic pollution studies in marine ecosystems. Researchers, however, concentrated more on plastic contamination in freshwater ecosystems, such as rivers, lakes, estuaries, and inland water, during phase III (2014–2020) (Kasavan et al., 2021).

Egypt is the largest user of polymers in Africa, consuming around 5.4 million tons of them per year (Ritchie & Roser, 2020). Aquatic ecosystems are being negatively impacted by Egypt’s excessive plastic usage, lack of waste management, and unrestricted dumping of plastic garbage (Sayed, Hamed, Badrey, & Soliman, 2021). Most water sources include plastics or microplastics that, directly or indirectly, enter aquatic systems before entering the bodies of aquatic animals, where they cause several negative effects that have been reported recently in several studies, and finally entering the food chain (Hamed et al., 2019, 2020; Sayed, Hamed, Badrey, & Soliman, 2021). The aquatic environment contains plastics in a wide range of sizes, from micrometers to meters (Van Cauwenberghe et al., 2015). Discarded plastic waste is gradually broken into smaller particles under the combined actions of physical abrasion and ultraviolet radiation (Fu et al., 2020). They were classified according to their size (microplastic ≤ 0.5 cm) which includes nanoplastics, which are particles with dimensions below 0.1 μm (1–100 nm) (Gigault et al., 2018), mesoplastic from 0.5 to 2.5 cm, macroplastic 2.5 cm–1 m, and megaplastic >1 m (Lusher et al., 2017), which have been adopted by UNEP (2020). Plastic contamination of surface water (Lahens et al., 2018), sediment (Renzi et al., 2020), and biota (Karlsson et al., 2017) has been reported from different places globally.

Most plastic pollution studies have concentrated on micro-, meso-, or macroplastics since research has shown that freshwater invertebrates and fish can ingest plastic particles. Very few of them reported all size ranges (Noik & Tuah, 2015). The Nile River is distinguished for its global significance and importance to Egypt as the longest river (6693 km) in the world (World Atlas, 2019), where it flows from south to north, passing through ten African countries before reaching the downstream country (Egypt), where its path starts from Aswan governorate in the far south, passing through eight governorates of Upper Egypt before flowing to Lower Egypt in Cairo, where it drains into the Mediterranean Sea through the Nile Delta.

It remains as the country’s principal supply of freshwater and provides almost all of its drinking and irrigation needs (Ali et al., 2014). It is the holy river of the ancient Egyptians, and the Nile’s historical dependency on agriculture, transportation, fishing, and tourism cannot be overstated in terms of how important a part it played in the development of ancient Egyptian civilizations (Dumont, 2009). The majority of plastic studies are still focused on the Red and Mediterranean seas, as well as some ecotoxicology laboratory experiments (Chatziparaskeva et al., 2022; El-Sayed et al., 2022; Hamed et al., 2019, 2020; Sayed et al., 2021,b; Shabaka*,* 2022), while the studies carried out on the Nile River so far are very few and were carried out only on the course of the Nile in Lower Egypt in Cairo and the Nile Delta (Khan et al., 2020; Shabaka et al., 2022).

Recently, the study of plastic has increased globally as well as in Egypt, and many methods have been used to estimate it. Numerous techniques have been created to precisely measure the riverine plastic flux or stock (van Emmerik & Schwarz, 2019). One commonly used method is the bridges’ visual counting method (Castro-Jiménez et al., 2019; Crosti et al., 2018). Observers stand on bridges and count the amount of visible floating and superficially submerged plastic for a predetermined amount of time in studies using this method, such as the pan-European RIMMEL project, which collected visual counting observations for over 40 rivers (González-Fernández et al., 2019). An active sampling of plastic debris is one of the most straightforward approaches to studying riverine plastic pollution (van Emmerik & Schwarz, 2019). This allows frequent and flexible measurements at different sites across the river. Considerable amounts of plastic can also be found in the sediment, in riverbanks, and in deeper layers of the water column. Sediment samples were, for example, analyzed for the Ombrone River in Italy (Guerranti et al., 2017), several rivers in Chile (Rech et al., 2015), and Germany (Kiessling et al., 2019; Morritt et al., 2014). Plastic exposure can have various physiological effects on growth, behavior, histopathology, metabolic changes, and reproductive success (Ahrendt et al., 2020; Rochman et al., 2014). Plastic and microplastics have been found in various animal species, including fish and invertebrates (Cole et al., 2011).

Despite the current rise in interest in researching plastic pollution in Egypt’s aquatic ecosystems, knowledge is still limited, notably on the Nile River. In particular, the plastic inputs call for further research. The current study’s objective is to document the occurrence and distribution of plastic in the water, sediment, fish, and crayfish from Upper Egypt’s Nile River.

## Materials and methods

### Study sites

**Fig. 1:**
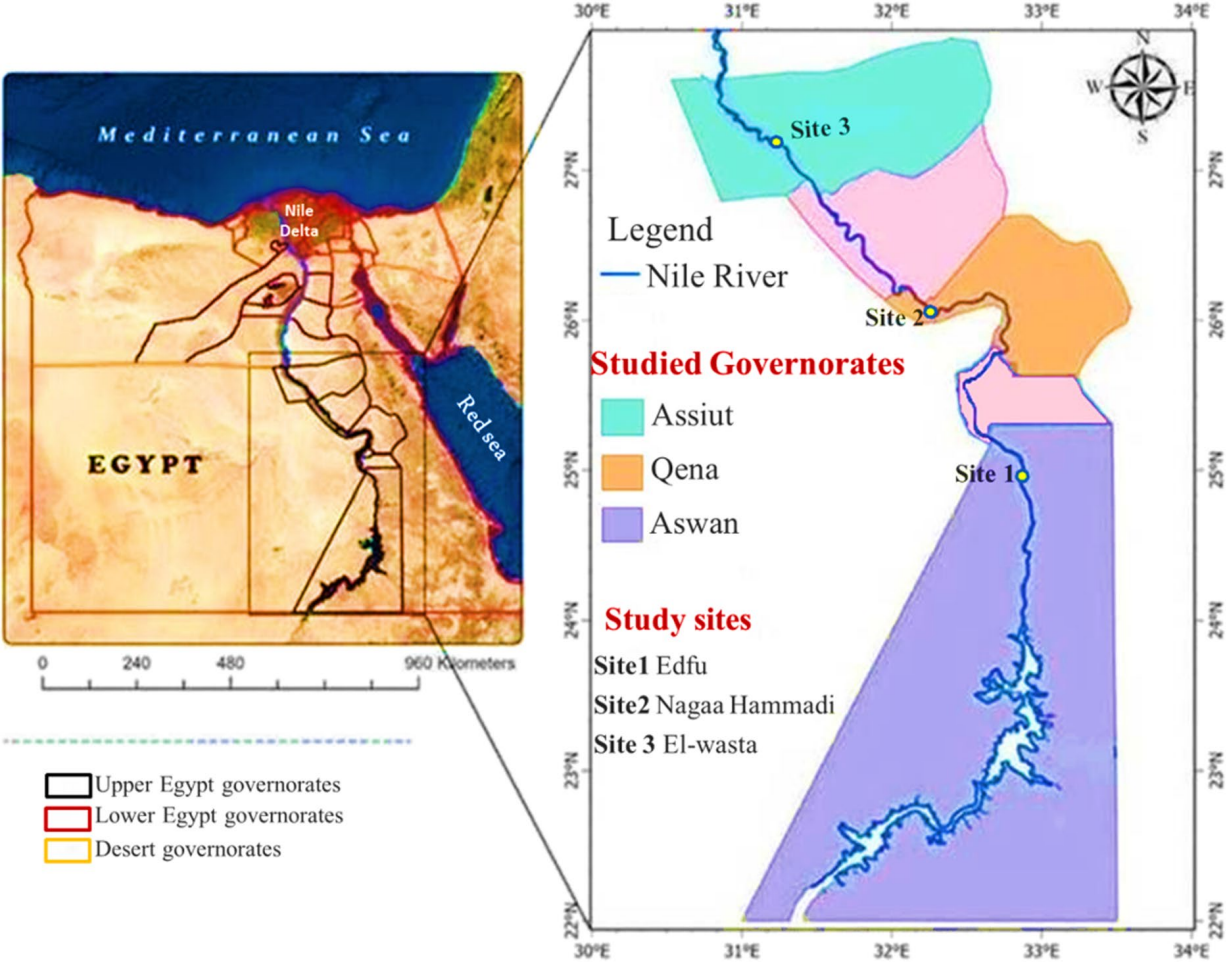
A map shows the study sites on the Nile River in Egypt

The current study was carried out during the winter of 2022 along the Nile River in three Upper Egypt governorates, Aswan, Qena, and Assiut. In each governorate, one site was chosen for the survey of plastics in water, sediment, fish, and invertebrate (Fig. 1). These sites were selected based on the varied urbanization and the diversity of anthropogenic activities: Edfu site (Aswan governorate) is a tourist site; Nagaa Hammadi site (Qena governorate) is an urban site, while El-wasta site (Assiut governorate) is a rural site.

### Sample collection and analysis

A one liter water sample (with three replicates) was collected using a labeled plastic bottle from each site. The samples were then sent to the lab for further analysis. Before being visually examined, each water sample (100 mL) was filtered through Whatman Nuclepore Hydrophilic Membrane filter paper (8 μm mesh size), and meso- (0.5–2.5 cm) and macroplastics (> 2.5 cm) were removed. Three sediment samples (1 kg each) were collected from each site using a Van Veen grab sampler (Sayed, Hamed, Badrey, Ismail, et al., 2021). These samples were packed tightly in labeled plastic bags and transported separately to the lab. About 100 g of sediment samples was placed in 500-mL beakers and then dried in an oven at 50 °C until a steady dry weight was noticed. A visual inspection was made of the sediments to remove plastic debris and meso- and macroplastics (> 0.5 cm), according to Mayoma et al. (2020). Three faunal species were collected from each site: two vertebrate species, Nile Tilapia *Oreochromis niloticus* (*N* = 7) and African catfish *Clarias gariepinus* (*N* = 6), and an invertebrate species, crayfish *Procamparus clarkii* (*N* = 35). The collected species were carefully placed in an ice box and transported to the lab for further processing. The alimentary canal and gills were removed and individually studied for each sample after their length and weight were measured. The animal species were collected using fishing nets and long lines. A dissecting binuclear microscope was used to visually investigate the meso- or macroplastics (> 0.5 cm) (Peters & Bratton, 2016). A digital camera was used to capture pictures of plastic objects at various magnifications.

### Statistical analysis

A database was created using Excel 2013 software to perform the first analysis using descriptive statistics, as well as to explain the differences in the average numbers and characteristics of meso- and macroplastics between all samples collected from the three sites. The data were presented as mean ± SD. The results which are related to the abundance of meso- and macroplastics in all samples collected from the three sites were subjected to a one-way analysis of variance (ANOVA) to test for significant differences between them. Data were analyzed using the SPSS program, version 16. Differences between means were compared using Duncan’s multiple range tests at a *p* < 0.05 level.

## Results

### The abundance and characteristics of meso- and macroplastic in water and sediment samples

**Fig. 2:**
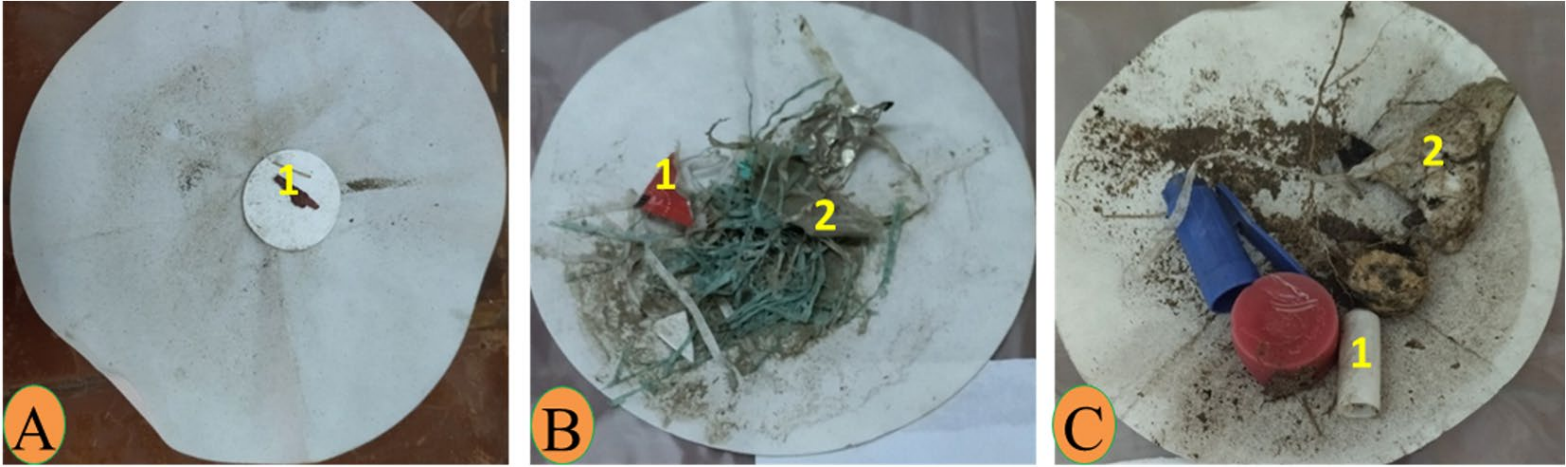
Photograph of meso- (1) and macroplastics (2) in water collected from the three sites: **A** Edfu, **B** Nagaa Hammadi, and **C** El-wasta

**Fig. 3:**
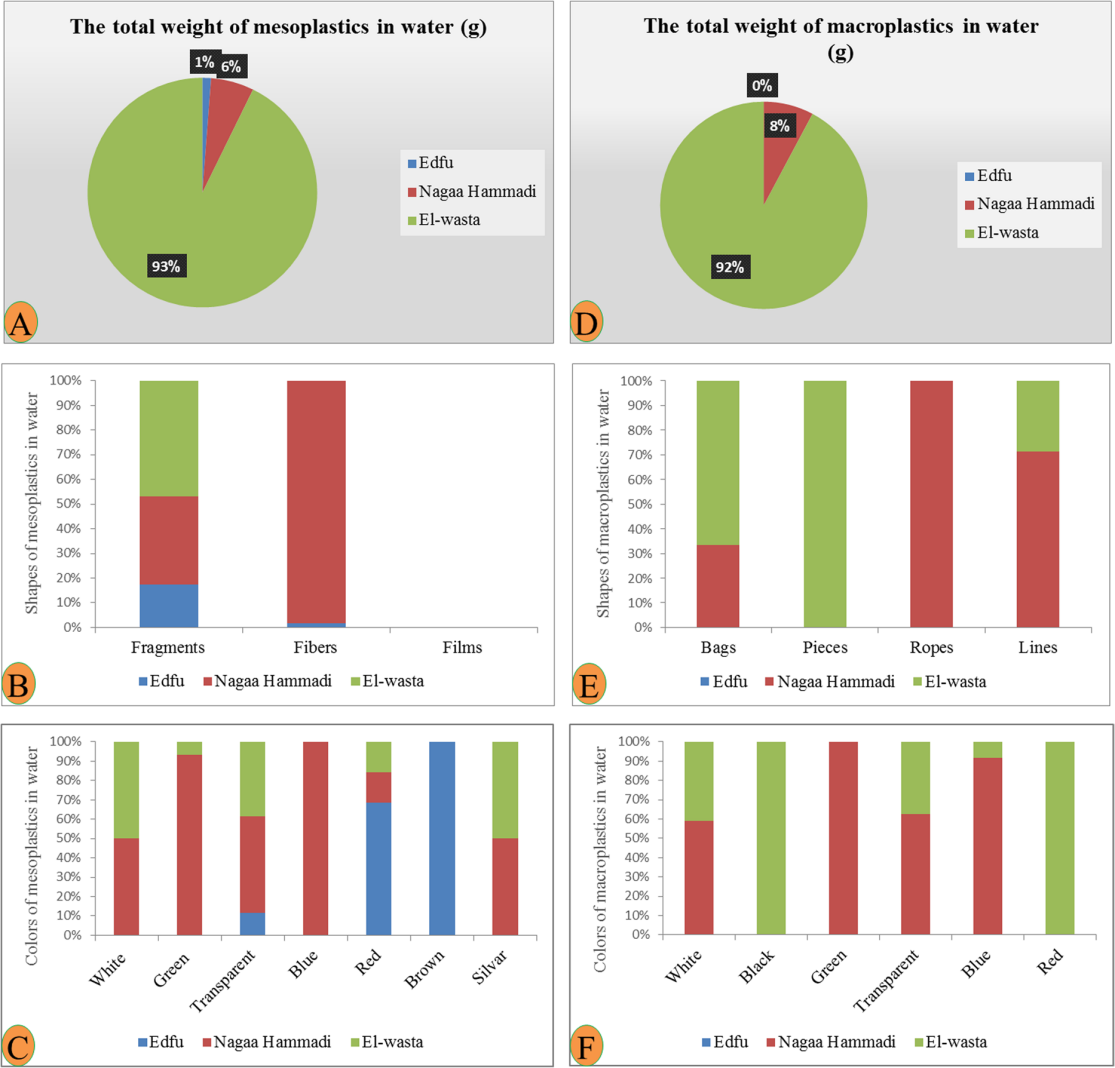
Characteristics of water mesoplastics: **A** total weight, **B** shapes, **C** color; and water macroplastics: **D** total weight, **E** shapes, and **F** color in the three sites

**Fig. 4:**
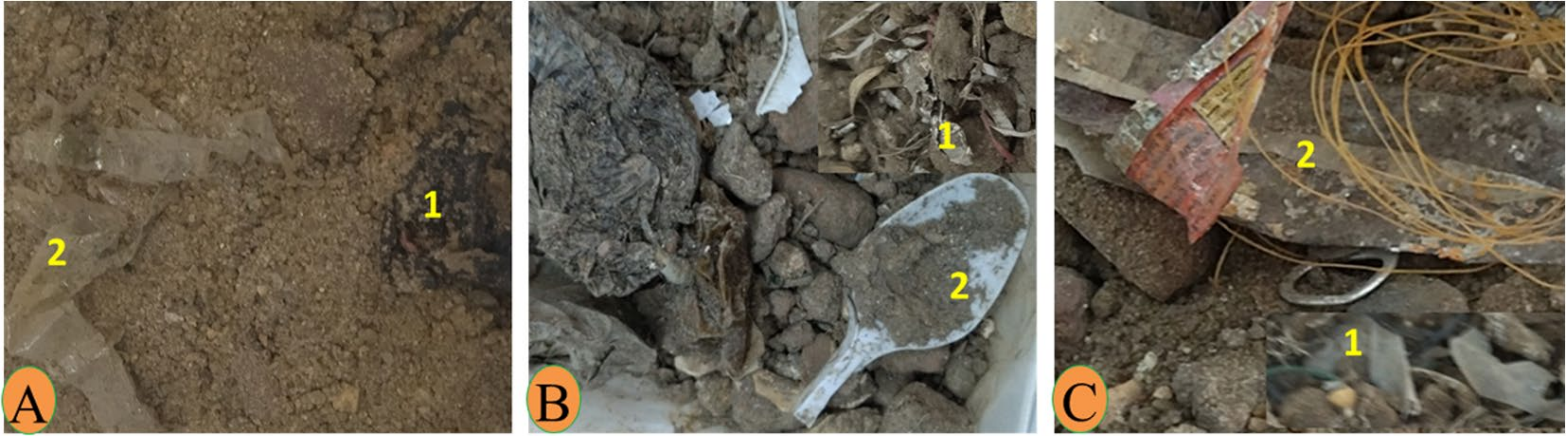
Photograph of mesoplastics (1) and macroplastics (2) in the sediments collected from the three sites: **A** Edfu, **B** Nagaa Hammadi, and **C** El-wasta

**Fig. 5:**
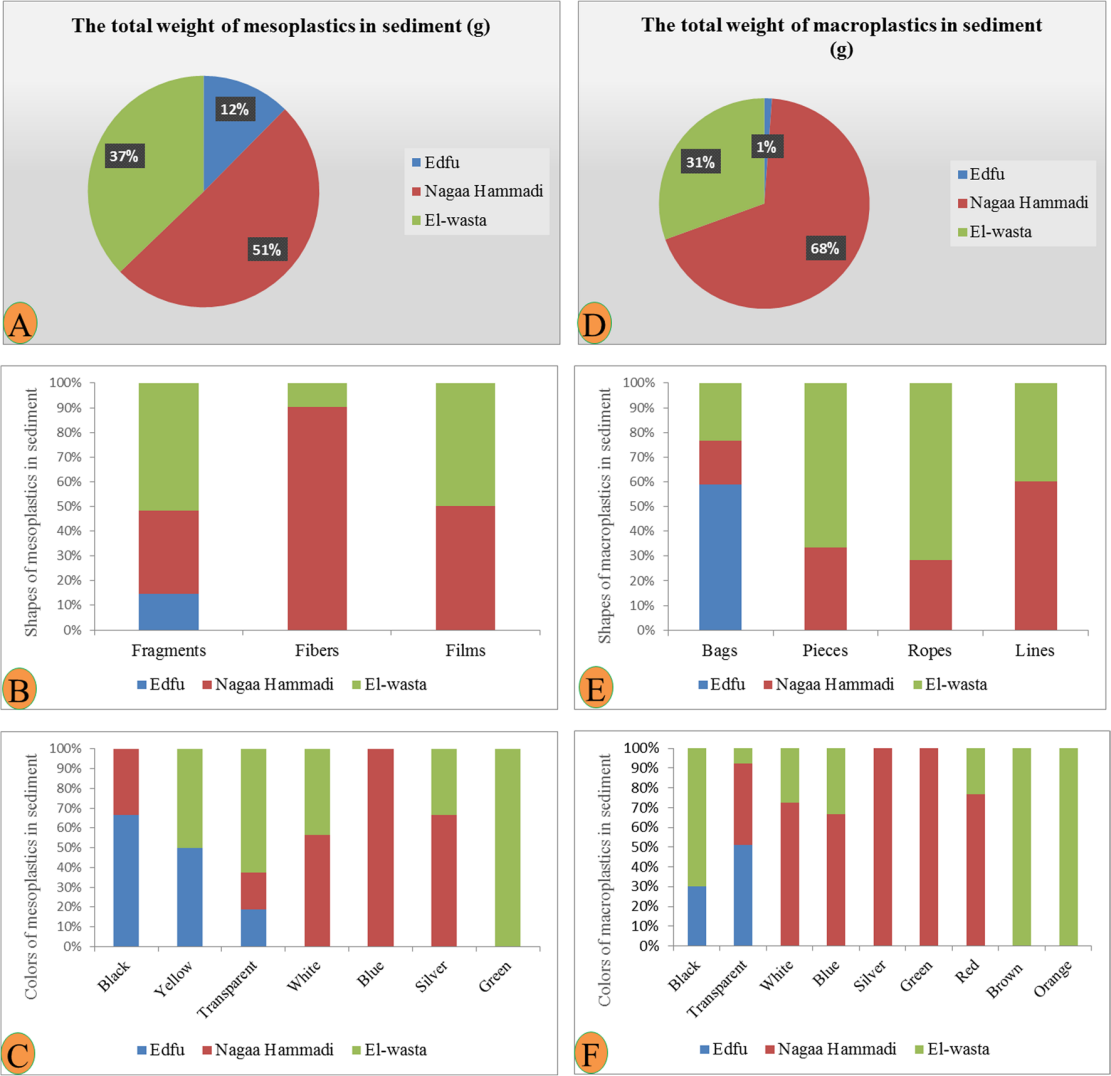
Characteristics of sediment mesoplastics: **A** total weight, **B** shapes, and **C** colors; and characteristics of sediment macroplastics: **D** total weight, **E** shapes, and **F** colors in the three sites

During the collection of water and sediment samples, different levels of abundance and composition of plastic waste were observed on the shores of the three sites. Nagaa Hammadi has the most abundant and composed site in plastic, followed by El-wasta, while Edfu was the least abundant and composed site in plastic. The average of the total numbers of meso- and macroplastic in water and sediment in the three sites (Edfu, Nagaa Hammadi, and El-wasta) is displayed in Table 1. The result revealed that meso- and macroplastics were higher in Nagaa Hammadi than in El-wasta and Edfu, with an average of meso- and macroplastics of 20.3 ± 3.5, 4.3 ± 2.5, and 2.0 ± 1.7 mesoplastic/100 mL and 14.3 ± 1.2, 6.0 ± 2.3, and 0 macroplastic/100 mL in water; and 9.0 ± 3.8, 5.6 ± 5.9, and 1.3 ± 1.3 mesoplastic/100 g and 10.6 ± 2.8, 10.6 ± 6.9, and 3.3 ± 0.5 macroplastic/100 g in sediment samples, respectively. Additionally, the results also showed the differences in the characteristics of the meso- and macroplastics that were discovered. El-wasta had the highest weight of meso- and macroplastics in water, followed by Nagaa Hammadi and Edfu. The shapes and colors of the meso- and macroplastics discovered in Nagaa Hammadi were the most varied (Figs. 2 and 3). On the other hand, the most dominant mesoplastic shape was fragments, followed by fibers, while in macroplastics, the most dominant shapes were bags and lines. As colors, the most dominant mesoplastic colors were transparent and red, while in macroplastics, the most dominant colors were white, transparent, and blue (Figs. 2 and 3). In sediment, the meso- and macroplastics discovered in the Nagaa Hammadi site had the highest weight, while those discovered in El-wasta and Nagaa Hammadi sites had a greater variety of shapes and colors than those discovered in the Edfu site (Figs. 4 and 5). On the other hand, the most dominant mesoplastic shape was fragments, while in macroplastics, the most dominant shape was bags. As colors, transparent was the most dominant color of meso- and macroplastics (Figs. 4 and 5).

**Table 1:** Shows the mean and standard deviation (mean ± SD) of the total number of meso- and macroplastics found in water and sediments at the three sites

Sampling sites	Water		Sediments	
	Mesoplastic (0.5–2.5 cm)	Macroplastic (> 2.5 cm)	Mesoplastic (0.5–2.5 cm)	Macroplastic (> 2.5 cm)
Edfu	2.0 ± 1.7^a^	0.0	1.3 ± 1.3^a^	3.3 ± 0.5^a^
Nagaa Hammadi	20.3 ± 3.5^b^	14.3 ± 1.2^a^	9.0 ± 3.8^b^	10.6 ± 2.8^b^
El-wasta	4.3 ± 2.5^ab^	6.0 ± 2.3^b^	5.6 ± 5.9^b^	10.6 ± 6.9^ab^

### The abundance and characteristics of meso- and macroplastics in the three freshwater animals

#### Nile tilapia Oreochromis niloticus

The number of fish that was collected from each site (Edfu, Nagaa Hammadi, and El-wasta) was seven fish, with an average length of 17.3, 20.08, and 23.3 cm and an average weight of 102.1, 178.5, and 122.1 g, respectively. The result shows that Nile tilapia collected from the three sites were not contaminated by meso- or macroplastics, neither in the alimentary canal nor in the gills (Table 2).

**Table 2:** Mean ± SD of the total number of mesoplastics in the three freshwater animals at the study sites

Sampling sites	Length (cm)			Weight (g)			Mesoplastic (0.5–2.5) (cm)				
	Edfu	Nagaa Hammadi	El-wasta	Edfu	Nagaa Hammadi	El-wasta	Edfu	Nagaa Hammadi		El-wasta	
							Alimentary canal	Alimentary canal	Gills	Alimentary canal	Gills
Nile tilapia	17.3 ± 2.6^a^	20.08 ± 2.08^b^	23.3 ± 13.9^c^	102.1 ± 62.9^a^	178.5 ± 61^c^	122.1 ± 64.3^b^	0.0	0.0	0.0	0.0	0.0
African catfish	36.3 ± 2.8^a^	39.4 ± 4.5^ab^	39.2 ± 9.3^a^	306.6 ± 64.2^a^	485 ± 146.9^b^	500.8 ± 390.0^c^	1.3 ± 1.1^a^	1.1 ± 1.1^a^	0.6 ± 1.1a	0.5 ± 0.7^b^	0.3 ± 0.4^b^
Crayfish	8.9 ± 0.9^a^	11.4 ± 1.1^b^	11.6 ± 1.04^ab^	12.2 ± 5.8^a^	28.7 ± 10.9^b^	28.7 ± 7.2^b^	0.1 ± 0.3^a^	0.2 ± 0.6^b^	0.0	0.1 ± 0.4^a^	0.0

#### African catfish Clarias gariepinus

**Fig. 6:**
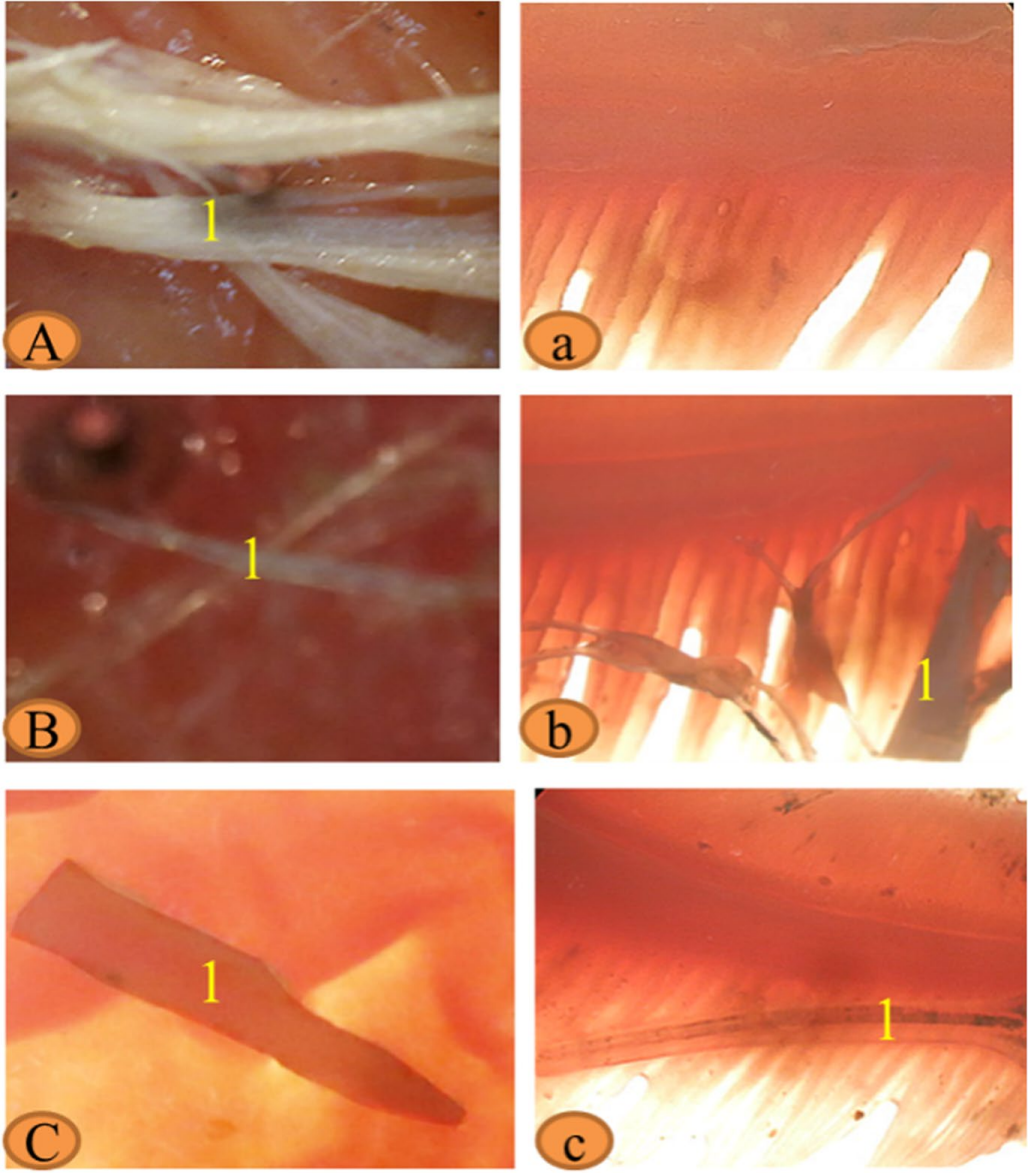
Binocular photograph of mesoplastic (1) in the alimentary canal (**A**, **B**, and **C**), and in gills (**a**, **b**, and **c**) in the African catfish *Clarias gariepinus* collected from the three sites **A**, **a** Edfu; **B**, **b** Nagaa Hammadi; and **C**, **c** El-wasta

**Fig. 7:**
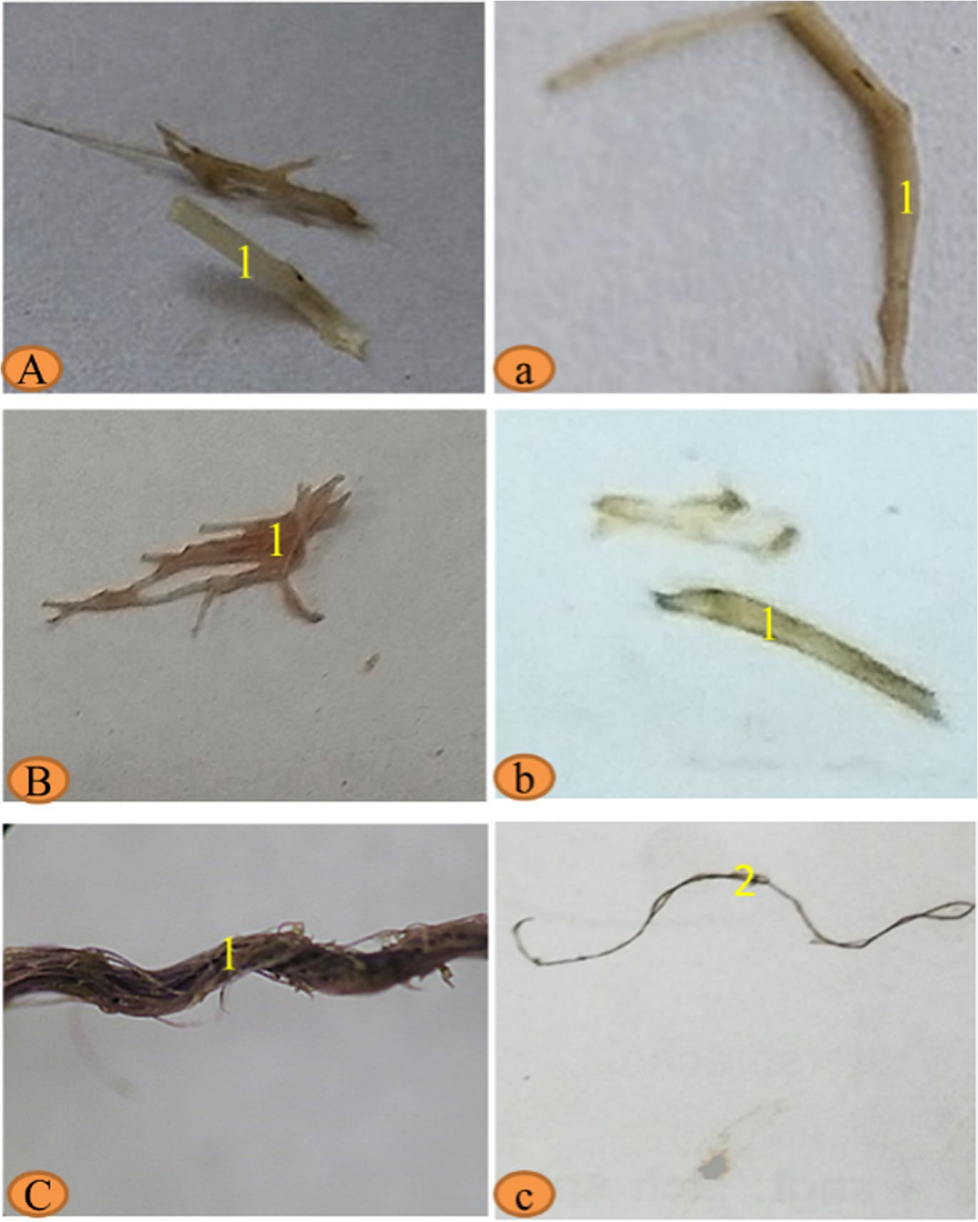
Photograph of isolated mesoplastic (1) and macroplastic (2) in the alimentary canal and gills of African catfish *Clarias gariepinus* collected from the three sites **A**, **a** Edfu; **B**, **b** Nagaa Hammadi; and **C**, **c** El-wasta

**Fig. 8:**
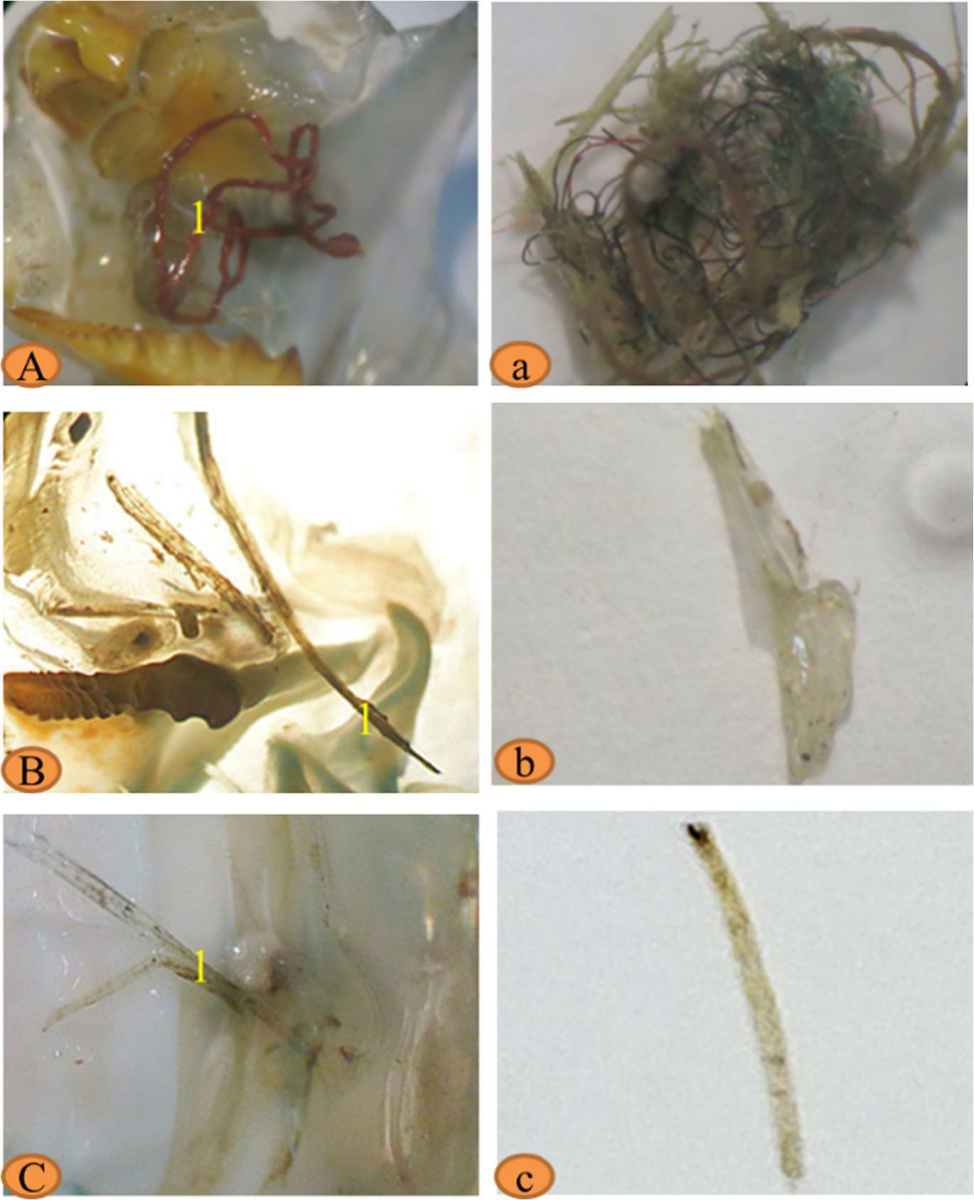
Binocular photograph of mesoplastic (**A**, **B**, and **C**) and isolated photograph mesoplastics (**a**, **b**, and **c**) in the alimentary canals of crayfish *Procamparus clarkii* collected from the three sites **A**, **a** Edfu; **B**, **b** Nagaa Hammadi; and **C**, **c** El-wasta

The numbers of fish that were collected from each site (Edfu, Nagaa Hammadi, and El-wasta) were six fish, with an average length of 36.3, 39.4, and 39.2 cm and average weight of 306.6, 485, and 500.8 g, respectively (Table 2). Our results showed that African catfish had the highest weight and abundance of meso- and macroplastics compared to the other species, and Edfu contains the largest number of mesoplastics in alimentary canals (1.3 mesoplastic/individual) followed by Nagaa Hammadi (1.1 mesoplastic/individual) and El-wasta (0.5 mesoplastic/individual), while in gills, Nagaa Hammadi contains the largest number of mesoplastics (0.6 mesoplastic/individual) followed by El-wasta (0.3 mesoplastic/individual) and no mesoplastics in the gills of *C. gariepinus* of Edfu (Table 2). Of the three species, macroplastics were only present in the alimentary canal of *C. gariepinus* collected from El-wasta. Mesoplastics had the highest weight in the alimentary canals and gills of African catfish in El-wasta. The most abundant mesoplastic shapes were film and fiber from the alimentary canals of all *C. gariepinus* in the three sites, and Nagaa Hammadi had a greater variety of shapes, while the most abundant mesoplastic shapes were from gill fiber and film in El-wasta and Nagaa Hammadi. On the other hand, from the macroplastic, only lines were detected (black color) in the alimentary canal of *C. gariepinus* collected from El-wasta. The most dominant mesoplastic color was white, followed by black, yellow, and orange in the alimentary canal, and El-wasta had a greater variety of colors. While in the gills, only two colors appeared from the mesoplastics: white and transparent in El-wasta and Nagaa Hammadi, and no mesoplastics were determined from gills collected from Edfu (Figs. 6, 7, and 8).

#### Crayfish Procamparus clarkii

**Fig. 9:**
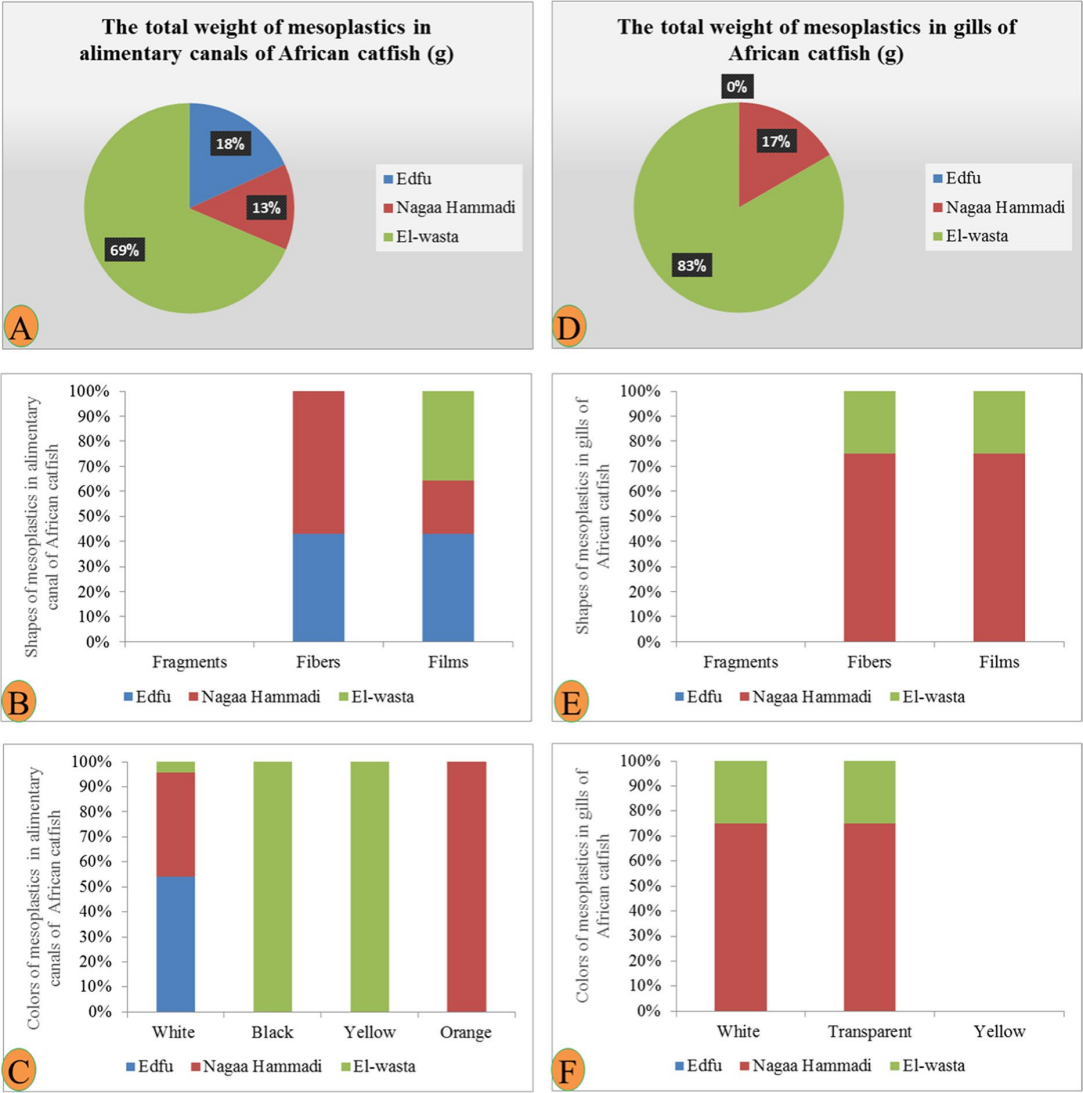
Characteristics of mesoplastics in the alimentary canals: **A** total weight, **B** shapes, and **C** colors; and characteristics of mesoplastic in the gills in African catfish, *Clarias gariepinus*, collected from three different sites: **D** total weight, **E** shapes, and **F** colors

**Fig. 10:**
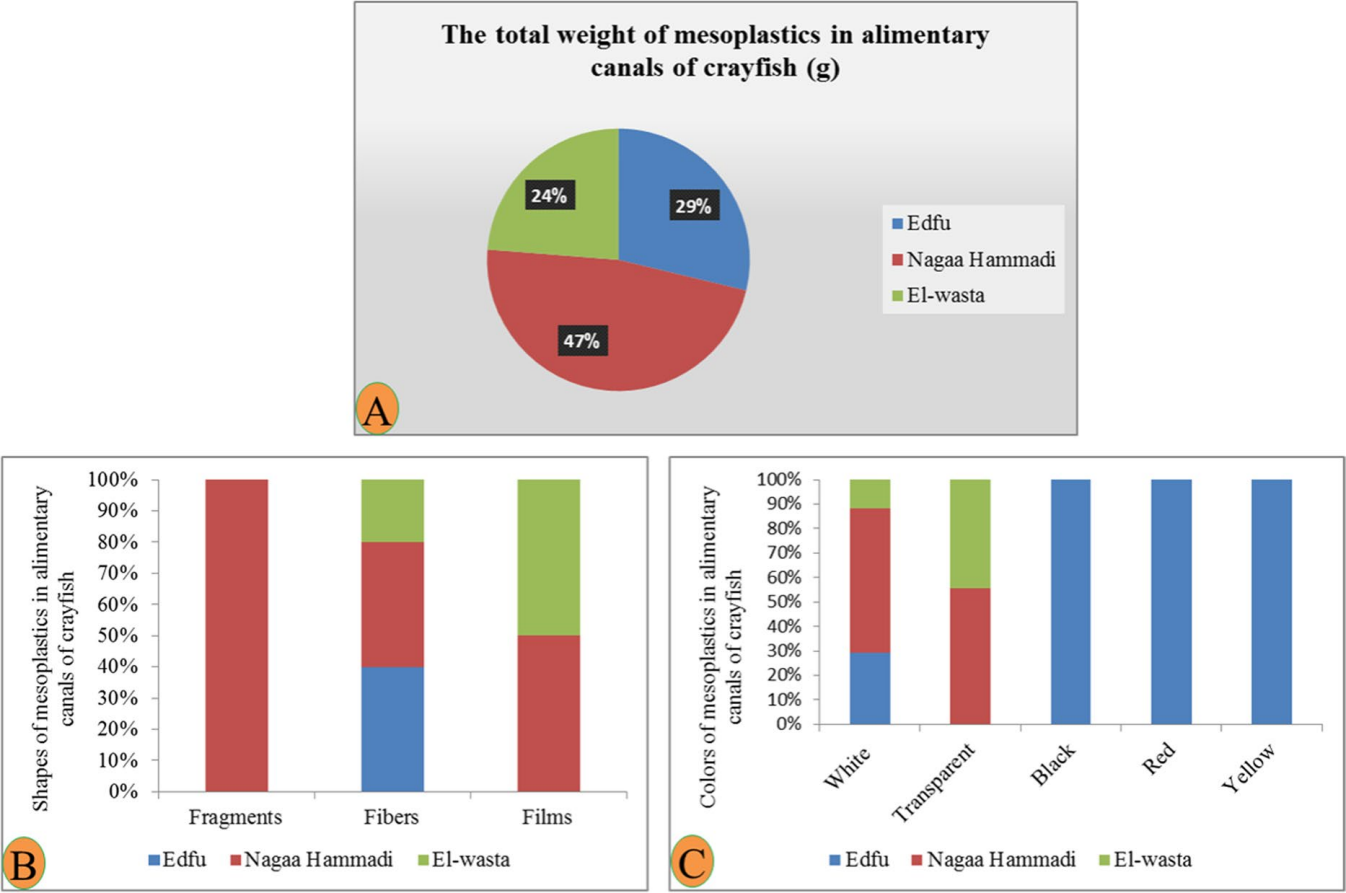
Characteristics of mesoplastics, **A** total weight, **B** shapes, and **C** colors, in the alimentary canal of crayfish, *Procamparus clarkii*, collected from the three sites

The numbers of *Procamparus clarkii* collected from each site (Edfu, Nagaa Hammadi, and El-wasta) were thirty-five, with average lengths of 8.9, 11.4, and 11.6 cm and average weights of 12.2, 28.7, and 28.7 g, respectively (Table 2). The macroplastics are not present in the alimentary canal and gills in all crayfish samples from the three sites. In addition, the results showed that mesoplastics are not present in all gills. Nagaa Hammadi contains the highest number and weight of mesoplastics in the alimentary canal for crayfish *P. clarkii*, followed by Edfu and El-wasta which contain an equal number of mesoplastics in the alimentary canal with an average number of mesoplastics of 0.2, 0.1, and 0.1 mesoplastic/individual, respectively (Table 2). The most abundant mesoplastic shapes were fibers, followed by film and fragments from the alimentary canals in the three sites, and Nagaa Hammadi had a greater variety of mesoplastic shapes than the two sites. On the other hand, the most dominant mesoplastic color was white, followed by transparent, then black, red, and yellow, and Edfu had a greater variety of mesoplastic colors than the two sites (Figs. 9 and 10).

## Discussion

The current study, which is regarded as a large-scale study, clarifies an important issue: the detection of meso- and macroplastics and a comparison of their abundance and characteristics in water, sediments, and some economically significant freshwater animals in the Nile River, the world’s longest river. Undoubtedly, the emergence of different pollutants on the Nile River is due to many sources, from natural to anthropogenic. The common pollution sources include industrial activities, urban development, tourism, fishing, shipping, resorts, and harbor activities. This claim is supported by the statistics that rivers are the primary transporters of plastic waste from terrestrial sources to the ocean (Lebreton et al., 2017; Zhang et al., 2017), especially via direct discharge, and that 80% of plastic waste in the aquatic environment originates from littering on land (Andrady, 2011). Plastics have also been identified as the primary component of anthropogenic trash on coastal beaches in European, North American, and South American clean-ups (Kordella et al., 2013). Also, Mayoma et al. (2020) found that about 80% of anthropogenic trash is often made up of plastic in Lake Malawi.

Egypt may have an impact on the amount of plastic waste entering the Nile River, which may subsequently affect the ecosystem. In addition, plastic bags, bottles, tires, other plastic wastes (such as fishing rope, lids, food wrappers, packaging material, cigarette butts, personal tools, and plastic pipes), and fishing gear are just a few examples of the various anthropogenic sources that contribute to the abundance of plastic waste along the Nile River shoreline. These plastics originated from human activities such as fishing, tourism, and urban garbage (Biginagwa et al., 2016). Meanwhile, wastewater treatment plants, cargo shipping, fishing, and human waste from beaches and urban runoff are important sources of plastic pollution (Cole et al., 2011; Peters & Bratton, 2016). Additionally, plastic pollution comes from sewage effluents, storm drains, winds, tide, and via recreational and commercial activities such as water sports and fishing (Borrelle et al., 2020; Schmaltz et al., 2020), causing a negative economic impact on tourism and posing serious biological risks to aquatic life (Avio et al., 2017). Once plastics reach freshwater, they may, for example, be trapped by instream structures (riverbanks, macrophytes, trees, and rocks), go with the water current to floodplain areas, or reach the sediment of contiguous sites (Peng et al., 2018; Weber & Opp, 2020).

The direct dumping of trash and waste into rivers is a long-standing custom in both urban and rural areas of the world and is only now restricted by law due to modernity and systems for managing liquid waste (Kalčíková et al., 2017). In general, meso- and macroplastics in the current study have been found in all waters, sediments, and vertebrate and invertebrate samples, with the exception of Nile tilapia. The Nagaa Hammadi site had the highest abundance of meso- and macroplastics in water, sediments, and animals followed by El-wasta. The reason that Nagaa Hammadi and El-wasta have more plastic in number, heavier weight, and a greater variety of shapes and colors than Edfu could be explained by the fact that synthetic polymers often weather into smaller particles over time (Li et al., 2018). Another explanation is that the Nile River flowed more slowly in these two sites since the samples were collected before the barrages that regulate the river flow in both sites, and possibly at Nagaa Hammadi due to the Nile River’s curvature in Qena before Nagaa Hammadi.

Plastic pollution has been reported in fresh water in many countries in different regions of the planet (Cera et al., 2020). The majority of these studies have been concentrated on plastic sizes less than 0.5 cm (microplastics), with a focus on meso- and macroplastics. Many authors previously observed the ingestion of plastics by animals (e.g., Cardozo et al., 2018; Lusher et al., 2013; Murray & Cowie, 2011). According to Evans et al. (2005), fish and invertebrate depend on their gills for respiration, which could become clogged or damaged by plastics, especially the small fragments, through obstruction and contamination. Our study recorded the appearance of plastic particles in bottom species and the absence of them in pelagic fishes. For comparison with related research, these findings were in consistent with those of a Chinese investigation (Jabeen et al., 2017) which found demersal species showed a significantly higher abundance of plastics than pelagic fish. This result is in contrast with the results reported for the fish from the North Sea and Baltic Sea (Rummel et al., 2016).

Here, we were limited to size-selected meso- and macroplastics > 0.5 cm in the current study. Additionally, smaller plastic items, including those in the nano-size range (less than 1 μm), may be able to penetrate fish tissue and cross intestinal barriers, allowing them to enter the human food chain (Revel et al., 2018). The appearance of larger meso- and macroplastics is less concerning in terms of human consumption since most aquatic animals, including those used in this study, are eaten after the removal of the digestive system and gills, but the presence of plastics in aquatic animals’ digestive systems and gills is a disturbing phenomenon that has been reported in many studies. In the present study, the absence of meso- and macroplastics in the alimentary canal and gills of Nile tilapia (*Oreochromis niloticus*) and their appearance in the African catfish (*Clarias gariepinus*) are due to the fact that their feeding habits have a significant impact on the amount of plastic waste they consume (Jabeen et al., 2017). Another interpretation is that larger fish (with larger mouths and guts, such as African catfish) may be able to ingest larger plastic particles (such as the size targeted in our study (> 0.5 cm)) than smaller fish (with smaller mouths and guts, such as Nile tilapia). As evidenced by the presence of macroplastics (> 0.5 cm) in the alimentary canal of *C. gariepinus* exclusively and the absence of these particles in the alimentary canals and gills of other animals, mesoplastics were not present in the gills of other animals. This was also supported in a study by Khan et al. (2020) on the Nile River in Cairo, where *Oreochromis niloticus* was one of the target species. It was discovered that 75.9% of the collected samples had microplastics (< 0.5 cm) in their digestive tract, which could be attributed to the smaller size of plastic targeted in this study versus the size targeted in our study. Another study supported by Jabeen et al. (2017) was conducted in China which found a higher abundance of microplastics than mesoplastics in all investigated fish. The results of our study may be higher than an Australian coastal study conducted by Puskic and Coghlan (2021) to detect minimal mesoplastics in Australian coastal reef fish. Of the 876 fish examined, the authors found that of 140 species (83 genera and 37 families), 12 individuals had mesoplastics in the gut.

Plastic waste in the water is rising, and mechanical degradation occurs when plastics physically come into contact with other polymers or objects. Other degradation mechanisms include heat and UV or photodegradation (Andrady, 2015). Plastics degrade more quickly on rivers and shores than they are in surface waters and sediments because they are exposed to higher temperatures and more UV radiation. Due to its slow rate, biodegradation is not thought to significantly contribute to plastic decomposition in aquatic settings, even though it does occur (Andrady, 2015). Since many species of invertebrates inhabit aquatic settings close to the sediment, they may be exposed to plastic pollution (Moreno & Callisto, 2006; Rosenberg & Resh, 1993). That explains the lower number of mesoplastics found in the alimentary canal of the crayfish *Procamparus clarkii*, their absence from the gills, and the absence of macroplastics. *C. gariepinus* alimentary canal contained a high concentration of mesoplastics, which are broken down into smaller particles and absorbed by aquatic animals due to the high temperature.

The varying sizes of the particles that these animals from various trophic guilds eat are probably related to behavioral variations. Despite the fact that all of these species are omnivores with distinct behavioral traits, *O. niloticus* eats plankton and smaller fish as part of its diet (FishBase, 2019). *C. gariepinus* occasionally feeds on insects, crabs, plankton, snails, and fish, but they have also been seen consuming young birds, and rotting meat, plants, and fruits (Teugels, 1986). *P. clarkii* consumes a mixture of edible and/or shelter-producing aquatic plants like Elodea and clever weeds like *Ceratophyllum demersum*, *Potamogeton nodosus*, and *Echhornia crassipes*. Additionally, *P. clarkii* eats living creatures, including earthworms and small fish. According to Ibrahim et al. (1995), this species can consume any kind of fish, depending on its capacity to grab its food.

## Conclusions

Meso- and macroplastics in the surface water, sediments, and fauna of the Egyptian Nile River were identified and quantified by sampling and visual examination, giving precise details on how frequently plastic waste is generated along the Nile River. The identification of the river Nile plastic debris may allow the decision-makers to consider how to reduce plastic-derived pollution along the river Nile. Taking into account the size of plastic pollution (meso- and macroplastic), plus their colors, could reduce the dangers that this pollution poses to river Nile fauna. The reduction of plastic consumption is one of the primary requirements for managing plastic debris in the river Nile and lowering its detrimental impacts on the freshwater ecosystem.

## Data Availability

The datasets generated during the study are available on reasonable request from the San Francisco Public Utilities Commission (SFPUC) and the corresponding author.
